# Interventions to improve cardiopulmonary resuscitation: a review of meta-analyses and future agenda

**DOI:** 10.1186/s13054-019-2495-5

**Published:** 2019-06-07

**Authors:** Athanasios Chalkias, John P. A. Ioannidis

**Affiliations:** 1 Department of Anesthesiology, University of Thessaly, Larisa, Greece; 2 Hellenic Society of Cardiopulmonary Resuscitation, Athens, Greece; 3 Meta-Research Innovation Center at Stanford (METRICS), Stanford University, Stanford, CA 94305 USA; 4 Department of Medicine, Stanford University, Stanford, CA 94305 USA; 5 Department of Health Research and Policy, Stanford University, Stanford, CA 94305 USA; 6 Department of Biomedical Data Science, Stanford University, Stanford, CA 94305 USA; 7 Department of Statistics, Stanford University, Stanford, CA 94305 USA

Hardly any other medical intervention is as directly relevant for life-and-death outcomes as cardiopulmonary resuscitation (CPR). One would have expected, therefore, extensive evidence from rigorous randomized controlled trials (RCTs) for fine-tuning best approaches that maximize CPR effectiveness. However, this is not the case. Professional guidelines reflect little tangible progress, and recommendations are not driven by strong effects seen in RCTs.

To map the landscape of meta-analyses of RCTs on CPR, we searched PubMed (April 8, 2019) for “cardiopulmonary resuscitation AND meta-analysis AND (randomized OR randomised).” We screened 114 retrieved items for meta-analyses of RCTs in real patients (not education or simulation/manikins), addressing aspects pertaining to CPR per se rather than interventions done afterwards (e.g., hypothermia) and using survival and/or neurologically intact survival as outcomes. Whenever multiple overlapping meta-analyses existed, we kept all of them if they were published after 2013, to examine consistency of results.

The available evidence (Table 1) suggests that we have a dearth of interventions that improve survival rates at hospital discharge and, even less so, neurological outcomes [1–12]. All benefits, if any, pertain to out-of-hospital cardiac arrest circumstances, while no new technology or improvement seems to work for in-hospital arrests. For out-of-hospital cardiac arrests, continuous (versus interrupted) chest compressions, epinephrine, and use of endotracheal tube intubation (versus supraglottic airway devices) may achieve modest increases in survival at hospital discharge. However, the lower 95% confidence intervals of the risk ratios in the most recent, inclusive meta-analyses on these interventions reach down to 1.00–1.02. Therefore, we cannot exclude that even these benefits are negligible or even non-existent. Survival with neurologically intact outcome is not conclusively increased by any of the interventions listed in the Table 1; epinephrine achieves a nominally statistically significant modest benefit over pooled control treatments, but this is less clear in separate comparisons against different control options. Epinephrine saves some patients who are admitted to the hospital, but they are not discharged neurologically intact. Other interventions also have disappointing results, e.g., no clear benefit is seen with mechanical devices for chest compression (they are even harmful for in-hospital cardiac arrest) and the order of chest compression versus defibrillation may not matter.

**Table 1:** Meta-analyses of randomized controlled trials with survival and neurologically intact survival as outcomes

	Comparison (setting)	*N* randomized	Outcome measures (timing) [*N*]	Relative risk (95% CI)	Heterogeneity
Chest compressions					
Meier et al. [1]	Chest compression-first vs. defibrillation-first (OHCA)	1503	Survival (HD) [*N* = 1503]	OR 1.10 (0.70–1.70)	*I*^2^ = 34%, *p* = 0.206
			CPC 1–2 (HD) [*N* = 402]	OR 1.02 (0.31–3.38)	*I*^2^ = 75%, *p* = 0.05
			Long-term survival (1 year) [*N* = 1301]	OR 1.38 (0.95–2.02)	*I*^2^ = 0.0%, *p* = 0.647^#^
Brooks et al. [2] and updated 2014 [3]	Mechanical vs. standard manual chest compressions (OHCA and IHCA)	868 and 1166^¥^	Survival (HA) [*N* = 164]	Not pooled	Studies not pooled
			CPC 1–2 (HD) [*N* = 767]	RR 0.41 (0.21–0.79)	Single study
			Survival (HD) [*N* = 1063]	Not pooled	Studies not pooled
Gates et al. [4]	Mechanical vs. standard manual chest compressions (OHCA)	12,206	Survival (HA) [*N* = 7208]	OR 0.95 (0.85–1.07)	*I*^2^ = 0.0%, *p* = 0.78
			Survival (HD or 30 days) [*N* = 12,206]	OR 0.89 (0.77–1.02)	*I*^2^ = 0.0%, *p* = 0.49
			CPC 1–2 or RS 0–3 (HD) [*N* = 12,206]	OR 0.76 (0.53–1.11)	*I*^2^ = 68%, *p* = 0.02
Tang et al. [5]	Mechanical vs. manual chest compressions (OHCA)	12,510	Survival (HA) [*N* = 12,510]	RR 0.94 (0.89–1.00)	*I*^2^ = 0.0%, *p* = 0.48
			Survival (HD) [*N* = 12,510]	RR 0.88 (0.78–0.99)	*I*^2^ = 27%, *p* = 0.24
			CPC 1–2 or RS 0–3 (HD) [*N* = 12,058]	RR 0.80 (0.61–1.04)	*I*^2^ = 65%, *p* = 0.04
			Long-term survival (≥ 6 months) [*N* = 7060]	RR 0.96 (0.79–1.16)	*I*^2^ = 16%, *p* = 0.28
Li et al. [6]	Mechanical vs. manual chest compression (OHCA and IHCA)	11,162	Survival (HA), OOH group [*N* = 9975]	RR 0.97 (0.91–1.04)	*I*^2^ = 60%, *p* = 0.015
			Survival (HD), OOH group [*N* = 4688]	RR 0.99 (0.82–1.18)	*I*^2^ = 71%, *p* = 0.004
			Survival (HD), IH group [*N* = 200]	RR 0.54 (0.29–0.98)	*I*^2^ = 0.0%, *p* = 0.825
			CPC 1–2 (HD), OOH group [*N* = 8885]	RR 1.11 (0.95–1.30)	*I*^2^ = 59%, *p* = 0.032
Zhan et al. [7]	Continuous (+/− rescue breathing) vs. interrupted chest compression with pauses for breaths (OHCA)	26,742^¶^	Survival (HA) [*N* = 520]	RR 1.18 (0.94–1.48)	Single study
			Survival (HD) [*N* = 3031]	RR 1.21 (1.01–1.46)	*I*^2^ = 0.0%, *p* = 0.68
			CPC 1–2 (HD) [*N* = 1286]	RR 1.25 (0.94–1.66)	Single study
Adrenaline					
Lin et al. [8]	SDA vs. placebo (OHCA)	12,246	Survival (HA) [*N* = 534]	RR 1.95 (1.34–2.84)	Single study
			Survival (HD) [*N* = 534]	RR 2.12 (0.75–6.02)	Single study
			CPC 1–2 (HD) [*N* = 534]	RR 1.73 (0.59–5.11)	Single study
	SDA vs. HDA (OHCA)		Survival (HA) [*N* = 5699]	RR 0.87 (0.76–1.00)	*I*^2^ = 34%, *p* = 0.21
			Survival (HD) [*N* = 5638]	RR 1.04 (0.76–1.42)	*I*^2^ = 0.0%, *p* = 0.66
			CPC 1–2 (HD) [*N* = 3883]	RR 1.20 (0.74–1.96)	*I*^2^ = 0.0%, *p* = 0.33
	SDA vs. vasopressin (OHCA)		Survival (HA) [*N* = 336]	Not pooled	Single study
			Survival (HD) [*N* = 336]	RR 0.68 (0.25–1.82)	Single study
			CPC 1–2 (HD) [*N* = 336]	RR 0.68 (0.25–1.82)	Single study
	SDA vs. vasopressin/adrenaline (OHCA)		Survival (HA) [*N* = 4877]	RR 0.88 (0.73–1.06)	*I*^2^ = 56%, *p* = 0.06
			Survival (HD) [*N* = 4877]	RR 1.00 (0.69–1.44)	*I*^2^ = 25%, *p* = 0.26
			CPC 1–2 (HD) [*N* = 4807]	RR 1.32 (0.88–1.98)	*I*^2^ = 0.0%, *p* = 0.85
Kempton et al. [9]	Epinephrine vs. placebo (OHCA)	17,635	Survival (HA) [*N* = 9511]	OR 2.52 (1.63–3.88)	*I*^2^ = 84%, *p* < 0.0001
			Survival (HD) [*N* = 9805]	OR 1.09 (0.48–2.47)	*I*^2^ = 77%, *p* = 0.0002
			CPC 1–2 or RS 0–3 (HD) [*N* = 9383]	OR 0.81 (0.34–1.96)	*I*^2^ = 83%, *p* = 0.0005
Finn et al. [10]	SDA vs. placebo (OHCA and IHCA)	21,704	Survival (HA) [*N* = 8489]	RR 2.51 (1.67–3.76)	*I*^2^ = 77%, *p* = 0.04
			Survival (HD) [*N* = 8538]	RR 1.44 (1.11–1.86)	*I*^2^ = 0.0%, *p* = 0.45
			Neurological outcome (HD) [*N* = 8535]	RR 1.21 (0.90–1.62)	*I*^2^ = 0.0%, *p* = 0.49
	SDA vs. HAD (OHCA and IHCA)		Survival (HA) [*N* = 5764]	RR 1.13 (1.03–1.24)	*I*^2^ = 0.0%, *p* = 0.42
			Survival (24 h) [*N* = 4179]	RR 1.04 (0.76–1.43)	*I*^2^ = 39%, *p* = 0.16
			Survival (HD) [*N* = 6274]	RR 1.10 (0.75–1.62)	*I*^2^ = 24%, *p* = 0.23
			Neurological outcome (HD) [*N* = 5803]	RR 0.91 (0.65–1.26)	*I*^2^ = 0.0%, *p* = 0.42
	SDA vs. vasopressin (OHCA and IHCA)		Survival (HA) [*N* = 1953]	RR 1.27 (1.04–1.54)	*I*^2^ = 27%, *p* = 0.25
			Survival (HD) [*N* = 2511]	RR 1.25 (0.84–1.85)	*I*^2^ = 29%, *p* = 0.22
			Neurological outcome (HD) [*N* = 2406]	RR 0.82 (0.54–1.25)	*I*^2^ = 0.0%, *p* = 0.46
	SDA vs. SDA + vasopressin (OHCA)		Survival (HA) [*N* = 3249]	RR 0.95 (0.83–1.08)	*I*^2^ = 0.0%, *p* = 0.55
			Survival (HD) [*N* = 3242]	RR 0.76 (0.47–1.22)	*I*^2^ = 0.0%, *p* = 0.57
			Neurological outcome (HD) [*N* = 2887]	RR 0.65 (0.33–1.31)	Single study
Vargas et al. [11]	Epinephrine vs. control (OHCA)	20,716	Survival (HA) [*N* = 20,306]	RR 1.02 (0.75–1.39)	*I*^2^ = 96.21%, *p* < 0.01
			Survival (HD) [*N* = 19,909]	RR 1.16 (1.00–1.35)	*I*^2^ = 0.0%, *p* = 0.49
			CPC 1–2 or similar (HD) [*N* = 18,458]^£^	RR 1.24 (1.05–1.48)	*I*^2^ = 0.0%, *p* = 0.94
Airway management					
White et al. [12]	Endotracheal tube intubation vs. supraglottic airway devices (OHCA)	539,146	Survival (HA) [*N* = 51,756]	OR 1.36 (1.12–1.66)	*I*^2^ = 91%, *p* = 0.002
			Survival (HD) [*N* = 440,564]	OR 1.28 (1.02–1.60)	*I*^2^ = 96%, *p* = 0.03
			CPC 1–2 or RS < 3 [HD] [*N* = 438,261]	OR 1.16 (0.94–1.41)	*I*^2^ = 91%, *p* = 0.16

*HA* hospital admission, *HD* hospital discharge, *RS* Rankin score, *OHCA* out-of-hospital cardiac arrest, *IHCA* in-hospital cardiac arrest, *SDA* standard dose adrenaline, *HAD* high-dose adrenaline

^¶^Randomized and quasi-randomized studies

^¥^From randomized controlled trials, cluster-randomized controlled trials, and quasi-randomized studies

^£^CPC 1–2, an overall performance category 1–2, a modified Rankin Scale score 1–2, and a normal or moderate disability

This rather disheartening evidence pertains largely to short-time follow-up. Longer-term outcomes are essential to make informed choices, but these data are rarely available from RCTs. One can try to supplement the evidence gap with observational datasets, and this is becoming increasingly convenient as large datasets become routinely available. However, for what are likely to be modest or subtle differences, it is unlikely that observational data will be sufficiently error-free to be conclusive. Many observational studies in this field claim sizeable survival differences, but their credibility is questionable—they need to be validated in carefully done RCTs [13]. For example, a highly cited observational study has found that endotracheal intubation is harmful for in-hospital arrest [14]. The availability of data on over 100,000 patients results in a very tight 95% confidence interval for neurological outcome and an astronomically low *p* value. However, this precision is misleading because potential bias may completely invalidate this conclusion.

In contrast to massive observational datasets, the RCTs done to-date and even their meta-analyses have usually had rather limited sample sizes. Clinically meaningful differences between the tested interventions may still have been missed, e.g., 20% relative risk differences in survival cannot be completely excluded for anything that has been tested to-date. This suggests that we need much larger RCTs in this field. Given that CPR is so commonly required, large simple trials should be feasible to do in large enough health care structures. It is important to instill in the future research agenda a strong element of pragmatism, so that the results would be more directly applicable to real-life circumstances. CPR is a good example where “point of care” randomization should be feasible without obtaining consent first given the nature of the intervention. Randomization should be the default option for CPR encounters if a protocol has been approved and set in place. RCTs with sample sizes in the tens of thousands of participants should be the goal.

A challenge in conducting such large-scale pragmatic RCTs is to avoid diluting the potential therapeutic effects by poor choices in the background management of the resuscitated patients. For example, an intervention may be effective by itself, but whatever benefit it produces may be lost if the patients undergo low-quality chest compressions or if they are then sub-optimally managed in the intensive care setting, e.g., improper choices are made for hypo- or hyper-ventilation. Meeting both pragmatism and some essential quality standards needs careful design and proper background training of the resuscitating and managing teams.

Another challenge is selecting the proper dose of various interventions to be tested. Several standard choices in the CPR ritual have little evidence to support that the dose, intensity, timing, or frequency used is optimized. For example, the standard dose of adrenalin (1 mg) is largely based on an experiment done over a century ago in 10-kg dogs, in which adrenaline was given at a dose of 0.1 mg/kg. While we have some randomized evidence on higher doses, we have no evidence on lower than standard doses. Timing may also be important. For example, another high-profile recent trial [15] administered epinephrine in patients who were largely “dead” (at 20 min post-arrest) and this may have affected its ability to be effective.

Finally, single interventions may have very limited efficacy and effectiveness, but their combination may manage to achieve a breakthrough in success rates. Testing this hypothesis would require running factorial trials, where two randomizations are performed concurrently. Then, one can assess both interventions as well as their joint effect in a statistically efficient manner.

CPR may save lives, and optimizing it should not be left to chance. A rigorous agenda of large pragmatic RCTs is long due. With simple design, the cost of these trials can be minimized, since data collection would pertain to only the most relevant information. Health care systems, insurances, and public agencies could make excellent investments in funding such trials.

## Data Availability

Not applicable.

## Abbreviations

CPR: Cardiopulmonary resuscitation
RCTs: Randomized controlled trials
